# Effect of Age and Lipoperoxidation in Rat and Human Adipose Tissue-Derived Stem Cells

**DOI:** 10.1155/2020/6473279

**Published:** 2020-12-09

**Authors:** Mario F. Muñoz, Sandro Argüelles, Francesco Marotta, Mario Barbagallo, Mercedes Cano, Antonio Ayala

**Affiliations:** ^1^Departamento de Bioquímica y Biología Molecular, Facultad de Farmacia, Universidad de Sevilla, Spain; ^2^Departamento de Fisiología, Facultad de Farmacia, Universidad de Sevilla, Spain; ^3^ReGenera R&D International for Aging Intervention & Vitality Therapeutics, San Babila Clinic, Milan, Italy; ^4^Department of Geriatrics and Internal Medicine, University of Palermo, Italy

## Abstract

A wide range of clinical applications in regenerative medicine were opened decades ago with the discovery of adult stem cells. Highly promising adult stem cells are mesenchymal stem/stromal cells derived from adipose tissue (ADSCs), primarily because of their abundance and accessibility. These cells have multipotent properties and have been used extensively to carry out autologous transplants. However, the biology of these cells is not entirely understood. Among other factors, the regeneration capacity of these cells will depend on both their capacity of proliferation/differentiation and the robustness of the biochemical pathways that allow them to survive under adverse conditions like those found in damaged tissues. The transcription factors, such as Nanog and Sox2, have been described as playing an important role in stem cell proliferation and differentiation. Also, the so-called longevity pathways, in which AMPK and SIRT1 proteins play a crucial role, are essential for cell homeostasis under stressful situations. These pathways act by inhibiting the translation through downregulation of elongation factor-2 (eEF2). In order to deepen knowledge of mesenchymal stem cell biology and which factors are determinant in the final therapeutic output, we evaluate in the present study the levels of all of these proteins in the ADSCs from humans and rats and how these levels are affected by aging and the oxidative environment. Due to the effect of aging and oxidative stress, our results suggest that before performing a cell therapy with ADSCs, several aspects reported in this study such as oxidative stress status and proliferation and differentiation capacity should be assessed on these cells. This would allow us to know the robustness of the transplanted cells and to predict the therapeutic result, especially in elder patients, where probably ADSCs do not carry out their biological functions in an optimal way.

## 1. Introduction

Adipose-derived stem/stromal cells (ADSCs) have proven to serve as an abundant, accessible, and rich source of mesenchymal stem/stromal cells (MSCs) [1, 2]. These cells have multipotent properties suitable for tissue engineering and regenerative medicine applications, including potential restoration of organ optimal functions that decline during aging [3, 4].

There is considerable evidence that deleterious molecular complex changes happen in all organisms during aging [5, 6]. Those changes also impact the regenerative capabilities of adult stem cells [7–10]. Although its causative role in aging remains to be demonstrated, oxidative stress is closely related to this process most of the time [11, 12]. Oxidative stress represents an unbalanced situation in which reactive oxygen species (ROS) generation exceeds antioxidant systems leading to tissue damage [11]. It is increasingly recognized that only unregulated levels of ROS are harmful, while regulated ROS production promotes essential signaling pathways, which regulate cell functions such as cell proliferation, differentiation, survival, and apoptosis [11, 13, 14].

Many aspects of stem cells (SCs) are affected by aging, which can limit their clinical applications. For instance, the number and quality of SCs decrease with donor age [15, 16] and affect the proliferation and differentiation potential both *in vitro* and *in vivo* [15, 17]. Transcription factors such as Nanog, Oct4, and Sox2 have been identified as essential regulators of proliferation and differentiation in SCs [18, 19], and their importance has even been reported in MSCs [20]. These transcription factors interact and regulate each other with their own expression [21]. The core of this network can also be deregulated during aging [20, 22], affecting the process of differentiation such as adipogenesis and osteogenesis in ADSCs [15, 23].

In addition to the aforementioned transcription factors, other proteins such as SIRT1 and AMP kinase (AMPK) have been described as important in the proliferation and differentiation capacity of stem cells. These proteins play an important role in the longevity and the capacity of tissue regeneration [24, 25]. AMPK is activated when the cellular ATP levels fall. In order to restore energy levels, AMPK inhibits anabolism (inhibits synthesis of proteins, cholesterol, and glycogen) and stimulates catabolic reactions from glucose and fatty acids during this energy stress. At the same time, it stimulates the recycling of cellular components (autophagy) [26]. SIRT1 is a NAD-dependent deacetylase with many functions in cellular metabolism, health, and aging [27], which is activated by caloric restriction and promotes cell survival. In fact, SIRT1 is able to decrease the levels of ROS by deacetylating and activating FOXO and PGC-1*α* [27].

Both AMPK and SIRT1 expression change with age and under oxidative stress conditions [24, 28, 29]. AMPK and SIRT1 have as target the elongation factor-2 (eEF2) [30, 31], which is the protein that moves the ribosome along the mRNA in the translation process. eEF2 can be regulated by multiple mechanisms [11, 32–34]. In general, those biochemical pathways that promote longevity act by inhibiting the translation through downregulation of eEF2 [30, 31].

Considering that (1) several proteins are involved in the maintenance of the self-renewal state and differentiation capacity of the stem cells, (2) these cells must perform their repairing function in adverse conditions. In this work, we evaluate some aspects of the biology of ADSCs that may affect their therapeutic capacity and/or their survival capabilities once they are transplanted into the site to be repaired. Here, we present how aging and oxidative stress, induced by cumene hydroperoxide (CH), can affect the viability and number of ADSCs, the levels of several proteins involved in the maintenance of the self-renewal state (Nanog and Sox2), the differentiation capacity, and the survival pathways (AMPK, SIRT1, and eEF2) in both rats and humans.

## 2. Material and Methods

### 2.1. Human Samples

300 ml of lipoaspirates was obtained after liposuction from the abdominal region of twelve female patients (body mass index between 24 and 29) that freely volunteered with different ages ranging from 25 to 48 years old (Table 1). Volunteers with pathologies were excluded. Samples were obtained from different aesthetic clinics under confidentiality and informed consent. All procedures were performed based on the regulations established by the Ethical Committee of Virgen del Rocío Hospital (Seville, Spain). Samples were stored at 4°C and processed within 24 h. The HeLa cell line (Life Technologies Invitrogen, Inc., Paisley, UK) was used as a human nonstem cell control for *in vitro* experiments and used at the same passage time after thawing. Cells were maintained in Dulbecco's modified Eagle's medium (DMEM; Life Technologies Invitrogen, Inc., Paisley, UK), supplemented with 10% fetal bovine serum, 50 U/ml penicillin G sodium, 50 U/ml streptomycin, and 2 mM L-glutamine.

### 2.2. Experimental Animals

All experiments were carried out according to the guidelines of the European Union Council (Directive 2010/63/UE) and to the Spanish regulations (BOE 34/11370, RD 53/2013) that were approved by the Ethics Committee of the University of Seville (# 19/03/2018/029). Male Wistar rats (250-700 g) were kept at a constant temperature of 22 ± 1°C and relative humidity of 60%, with a light-dark cycle of 12 h and free access to food and water.

In order to study the effect of aging, three groups of four animals each were used: 2-, 9-, and 24-month-old rats. To evaluate the effect of oxidative stress, one-month-old rats were divided randomly into three groups of five animals each, which were intraperitoneally injected with NaCl 0.9% (control) and 40 and 80 mg/kg/day of CH, respectively, for 30 days [34, 35].

### 2.3. Rat Adipose Stromal Vascular Fraction Isolation

Animals were anesthetized with ketamine/xylazine (100/12.5 mg/kg). Subcutaneous adipose tissue from the inguinal region was obtained and weighted in sterile conditions. The tissue was washed with sterile phosphate-buffered saline (PBS) and was enzymatically processed by collagenase (Collagenase NB 4G, Serva, Iceland, NY, USA) solution (0.9 units/ml) incubated at 37°C for 150 min on an orbital shaker. Then, an equal volume of “stop medium” (Dulbecco's modified Eagle's medium (DMEM)) (Sigma-Aldrich, Saint Louis, MO, USA) with 20% fetal bovine serum (FBS) (Sigma-Aldrich) was added; then, samples were centrifuged at 600 × *g* for 10 min at 4°C. The pellet was resuspended in culture media (DMEM+10% FBS+1% penicillin-streptomycin), and the cell suspension was filtered through a 40 *μ*m pore-size filter to obtain the stromal vascular fraction (SVF).

### 2.4. Human Adipose Stromal Vascular Fraction Isolation

Lipoaspirates were washed with PBS and enzymatically processed by collagenase (Collagenase NB 4G, Serva, Iceland, NY, USA) solution incubated (0.3 units/ml) at 37°C for 120 min in an orbital shaker. Then, an equal volume of “stop medium” (DMEM) with 20% FBS was added and samples were centrifuged at 600 × *g* for 10 min at 4°C. The pellet was resuspended in DMEM+10% FBS+1% penicillin-streptomycin, and the cell suspension was filtered through a 100 *μ*m pore-size filter. This last step was repeated but using a 40 *μ*m pore-size filter to obtain human SVF.

### 2.5. Rat and Human ADSC Isolation

In order to perform the extraction of the ADSCs according to guidelines established by the International Society for Cellular Therapy (ISCT), we first performed a magnetic separation based on MACS© technology (Magnetic Cell Sorting, Miltenyi Biotec, Bergisch Gladbach, Germany). This procedure is based on the use of the so-called “microbeads” which are magnetic microparticles associated with antibodies that specifically recognize antigens on the surface of stem cells [36, 37]. The CD271 (low-affinity nerve growth factor receptor (LNGFR)) APC-conjugated antibody (Miltenyi Biotec, Bergisch Gladbach, Germany) with secondary anti-APC microbeads (Miltenyi Biotec, Bergisch Gladbach, Germany) was chosen to obtain human ADSCs (hADSCs) [38–40]. Because the homologous CD271 marker has not been described in rats, we used CD90.1 (thymocyte differentiation antigen-1.1, a mouse monoclonal antibody that reacts with rat CD90) microbead antibody (Miltenyi Biotec, Bergisch Gladbach, Germany) to obtain rat ADSCs (rADSCs) [41, 42]. After magnetic separation, cells were centrifuged, resuspended, and cultured in DMEM+10% FBS+1% penicillin-streptomycin for rADSCs and StemMACS MSC expansion media (Miltenyi Biotec, Bergisch Gladbach, Germany) for hADSCs. Finally, cells were incubated and maintained in culture at 37°C in 5% CO_2_. Because of the loss of pluripotency in hMSCs and rADSCs during culture, we limited the expansion time to four passages. Thus, all samples were five cell passages old when assays were assessed.

### 2.6. Phenotyping Rat and Human ADSCs by Flow Cytometry

To verify the presence of surface markers expressed by cells such as ISCT suggests, we used a human MSC phenotype kit (Miltenyi Biotec, Bergisch Gladbach, Germany) for hADSCs, which contains the CD14-PerCP, CD20-PerCP, CD34-PerCP, CD45-PerCP, CD73-APC, CD90-FITC, and CD105-PE monoclonal antibodies. hADSCs were detached from culture, and 1 × 10^5^ cells were treated following the manufacturer's instructions. For rADSC phenotyping, we used the monoclonal antibodies labeled with mouse anti-CD34-PE (Santa Cruz Biotechnology, Santa Cruz, CA, USA), CD45-PE (BD Pharmingen, Dallas, TX, USA), CD11b-PE (BioLegend, San Diego, CA, USA), and CD29-APC and CD90.1-FITC (Miltenyi Biotec, Bergisch Gladbach, Germany). The cytometry analysis was carried out in a FC500 Cytometer (Beckman Coulter, Pasadena, CA, USA), and the data was analyzed using Summit 2.0 software. Voltage and color compensations were previously set up.

### 2.7. Rat and Human ADSC Differentiation

The differentiation capacity was assessed following the manufacturer's instructions for commercial differentiation kits. For rADSCs, cells were cultured in a differentiation medium (StemXVivo Osteogenic/Adipogenic Media-R&D Systems, Minneapolis, MI, USA) with adipogenic or osteogenic supplements contained in the kit. Adipocytes were detected by Oil Red O (Sigma-Aldrich, Saint Louis, MO, USA) staining and osteoblasts by NBT/BCIP (Sigma-Aldrich, Saint Louis, MO, USA) staining. To differentiate into chondrocytes, 2.5 × 10^5^ cells were cultured in DMEM/F-12 media (Sigma-Aldrich, Saint Louis, MO, USA) plus chondrogenic supplements (StemXVivo Chondrogenic Supplement-R&D Systems) in the bottom of a 15 ml conical tube. The cell pellet formed a rounded ball approximately 1-2 mm in diameter on the 3^rd^ day. On the 14-28^th^ days, the pellet was fixed in 4% paraformaldehyde and cut into 10 *μ*m sections. They were treated with chondrocyte-specific primary antibody anti-aggrecan (R&D Systems, Minneapolis, MI, USA) and anti-rabbit Alexa Fluor 488 (Thermo Fisher Scientific, Waltham, MS, USA) as a secondary antibody. For hADSCs, cells were cultured in StemMACS AdipoDiff media and StemMACS OsteoDiff media (Miltenyi Biotec, Bergisch Gladbach, Germany). The procedure of detection was similar to that of rADSCs.

### 2.8. Cell Number and Viability

After immunomagnetic isolation, cells were counted in a cell counter (Countess Invitrogen, Thermo Fisher Scientific, Inc., Paisley, UK). The percentage of viability was measured by trypan blue. Each sample was measured by technical triplicates.

### 2.9. Growth Curves

The growth characteristics were assessed in rADSCs isolated from both animals of different ages and treated with CH. The same number of cells (5 × 10^4^ cells/well) from each sample was cultured in duplicate in a 6-well plate. At different times (days 3, 5, and 7), cells were harvested and counted as indicated above. These cells reached a lag phase on the 2^nd^-3^rd^ days, a log phase on the 3^rd^-6^th^ days, and a plateau phase after the 7^th^ day (at a density of 5 × 10^3^/cm^2^ in a 6-well plate).

### 2.10. Tissue and Cell Homogenization

Hepatic tissue from the same animals was homogenized in 10 mM N-2-hydroxyethylpiperazine-*N*′-2-ethanesulfonic acid (HEPES), pH 7.0, and 0.2 mM phenylmethylsulfonyl fluoride (PMSF) buffer. The homogenate was centrifuged at 800×*g* for 10 min. The supernatant was centrifuged at 12,000×*g* for 20 min at 4°C, and the pellet was discarded. Cells were lysed in RIPA buffer (20 mM Tris-HCl, 150 mM NaCl, 1 mM EDTA, 1 mM EGTA, 1% NP-40, 1% sodium deoxycholate, 2.5 mM sodium pyrophosphate, and 1 mM sodium orthovanadate) containing protease inhibitors. The homogenized cells were centrifuged at 12,000×*g* for 20 min at 4°C, and the supernatant was collected.

### 2.11. 4-Hydroxynonenal Adduct Analysis

Protein extracts from the liver and cells were measured by enzyme-linked immunosorbent assay (ELISA) to detect 4-hydroxynonenal (4-HNE) adducts in the samples, following the manufacturer's instructions (OxiSelect© HNE-His, Cell Biolabs, San Diego, CA, USA).

### 2.12. Quantification of Differentiation

The total number of adipose cells was counted in duplicate from each sample, using five consistent regions within each well. Multiple fields of view (×200 magnification) were obtained from each of these regions, starting from the center of the well and traveling out to the periphery, but not near the walls to avoid the meniscus effect [43]. Values from these regions were averaged to give the mean differentiation level for each sample and were expressed in percentage, the control group being 100% of the adipogenic differentiation. In hADSCs, adipogenesis was measured by quantifying the levels of glycerol-3-phosphate dehydrogenase (GPDH) (Sigma-Aldrich, Saint Louis, MO, USA) by Western blot (WB) [44–46]. To assess the levels of calcium deposit after osteogenic differentiation, the osteoblast matrix was demineralized by the addition of 500 *μ*l of 0.6 N HCl and incubated overnight at 37°C. Solutions were collected and centrifuged for 5 min at 250×*g*. Calcium concentrations in the supernatants were determined colorimetrically (QuantiChrom Calcium Assay Kit, BioAssay Systems, Hayward, CA) according to the manufacturer's instructions, by measuring the absorbance at 610 nm, using a plate reader (Asys UVM340, Biochrom, Cambourne, UK).

### 2.13. Western Blotting

The protein content of the samples was estimated with the DC Protein Assay Kit (Bio-Rad, Hercules, CA, USA). Protein samples were separated by SDS-PAGE (10% acrylamide) and transferred to the nitrocellulose membrane (Bio-Rad, Hercules, CA, USA) at 120 V for 1 h. The membranes were incubated with a blocking buffer (5% dry milk in 20 mM Tris-HCl, pH 7.5, 500 mM NaCl, and 0.05% Tween 20) for 1 h at room temperature. Membranes were incubated overnight at 4°C in a blocking solution containing the following antibodies: eEF2 (1 : 1000), AMPK*α* (1 : 1000), SIRT1 (1 : 1000) (Cell Signaling Technology, Danvers, MA, USA), Sox2 (1 : 500), Nanog (1 : 500) (Abnova, Taipei, Taiwan), GPDH (1 : 1000), GAPDH, *β*-actin, and *α*-tubulin (1 : 3000) (Sigma-Aldrich, Saint Louis, MO, USA). After incubation, the membranes were washed in 20 mM Tris-HCl, pH 7.5, 500 mM NaCl, and 0.05% Tween 20 and incubated with peroxidase-conjugated anti-immunoglobulin secondary antibodies. The proteins were visualized using chemiluminescence reagents (Advansta, Menlo Park, CA, USA). The bands were analyzed by densitometry using ImageJ analysis software (NIH). GAPDH and *α*-tubulin were used as loading controls. Some membranes were reused with Restore® Western Blot Stripping Buffer (Thermo Fisher Scientific, Waltham, MA, USA) following the manufacturer's instructions.

### 2.14. MTS/PMS Viability Assay

Cells were cultured in a 96-well plate at 1.5 × 10^4^ cells/well density overnight. Then, they were exposed to different concentrations of CH (0-120 *μ*M) for 3 h. After treatment, the medium was removed and washed twice with PBS. 120 *μ*l of the culture medium with MTS/PMS (CellTiter 96, Promega, Madison, WI, USA) was added. Two hours later, absorbance was measured in a plate reader (*λ*_max_ = 490 nm).

### 2.15. Statistical Analysis

Data are shown as the mean ± SEM. One-way ANOVA, followed by Tukey's test, was used to compare more than two groups, and Pearson's test was used to bivariate correlations. All analyses were performed in SPSS 21 software (IBM, Armonk, NY, USA). A value of *p* ≤ 0.05 was considered significant.

## 3. Results

### 3.1. Characterization of ADSCs

Mesenchymal and Tissue Stem Cell Committee of the International Society for Cellular Therapy (ISCT) proposed a set of standard criteria to define this population of cells [1, 47]. These criteria are as follows: adherence to plastic under standard culture conditions; “*in vitro*” differentiation into osteoblasts, adipocytes, and chondroblasts; specific surface antigen expression in which 95% of the cells express the antigens CD105, CD73, and CD90.1; and absence (<3% positive) of hematopoietic lineage antigens CD45, CD34, CD14 or CD11b, CD79a or CD19, and HLA-DR. In the case of murine models, these criteria have not been established yet, though many authors replicate the criteria established for human cells [41, 48, 49]). In this work, we confirmed that both the rat and human ADSCs meet these criteria. Both rADSCs and hADSCs showed adherence to the plastic and absence of cells in suspension under standard culture conditions (Supplementary Figures 1A and 2A, respectively).

For rADSCs, using flow cytometry, we found that 98.0 ± 0.9% of the events were positive for MSC markers such as CD29 and CD90.1. Less than 3% of all events showed positive expression for any of the hematopoietic markers CD11b, CD34, and CD45 (Supplementary Figure 1B). The differentiation of rADSCs into osteoblasts, adipocytes, and chondroblasts was analyzed *in vitro*. We observed the intracellular accumulation of lipids detected by Oil Red O staining after adipogenic differentiation; high expression of phosphate alkaline showed osteogenic differentiation, and positive anti-aggrecan immunofluorescence indicated chondrogenic differentiation (Supplementary Figure 1C).

The same procedures were performed with hADSCs to assess phenotyping and differentiation. The flow cytometry analysis showed that the number of events in the double-positive region for CD90/CD105 and CD90/CD73 was 99.67 ± 0.32% and 99.69 ± 0.20%, respectively. The expression of CD14, CD20, CD34, and CD45 as hematopoietic markers was less than 0.5% (Supplementary Figure 2B). In addition, hADSCs from patients of different ages were differentiated into osteoblasts and adipocytes (Supplementary Figure 2C). Taking together, these results indicate that both the rat and human cells isolated from adipose tissue can be considered MSCs.

### 3.2. Effect of Age and *In Vivo* CH Treatment on 4-HNE-Protein Adduct Formation in the Liver and rADSCs

**Figure 1:**
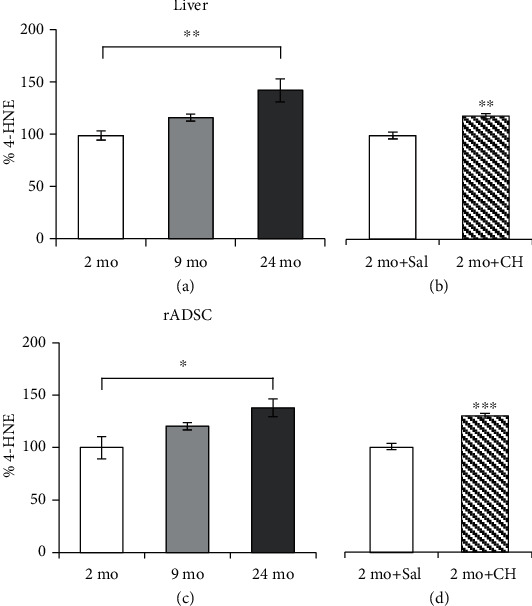
Effect of age and *in vivo* CH treatment on 4-HNE-protein adduct formation in the liver and rADSCs. Quantification of 4-HNE levels measured by ELISA in the liver (a, b) and rADSCs (c, d) obtained from 2-, 9-, and 24-month-old rats (*n* = 4) (a, c) and from 2-month-old rats treated with 40 mg/kg/day of CH (2 mo+CH) or saline (2 mo+Sal) (*n* = 4) (b, d). Values are the mean (%) ± SEM expressed as percentages with respect to the value of the control group (100%). One-way ANOVA followed by Tukey's test was performed for multiple comparisons. ^∗^*p* < 0.05 and ^∗∗^*p* < 0.01 (a, c). One-way ANOVA was performed for comparison of two groups. ^∗∗^*p* < 0.01 and ^∗∗∗^*p* < 0.001 (b, d).

As we reported in previous studies, age and CH treatment can induce 4-HNE-protein adduct formation in the liver both *in vitro* and *in vivo* [33]. To ensure that the age of the rats we used in these experiments and CH treatment induced LP, the levels of the 4-HNE-protein adduct were determined in both the liver and the rADSCs of rats of different ages and treated with CH. In the liver, we found a significant increase of 42% of the 4-HNE adduct in the 24-month-old group with respect to the levels found in the 2-month-old group (Figure 1(a)). No significant differences were found between the other age groups. In livers from animals treated with 40 mg/kg/day of CH, we found an increase of 18.7% of the 4-HNE adduct, compared with the control group (Figure 1(b)).

In rADSCs, the increase of 4-HNE-protein adduct formation was similar to that found in liver samples: 38% increase in the 24-month-old group with respect to young rats (Figure 1(c)). When animals were treated with CH, the amount of adduct formation between 4-HNE and proteins of ADSCs was slightly higher than in the liver (Figure 1(d)).

### 3.3. Effect of Age and *In Vivo* CH Treatment on the Number, Viability, and Growth Curve of rADSCs

**Figure 2:**
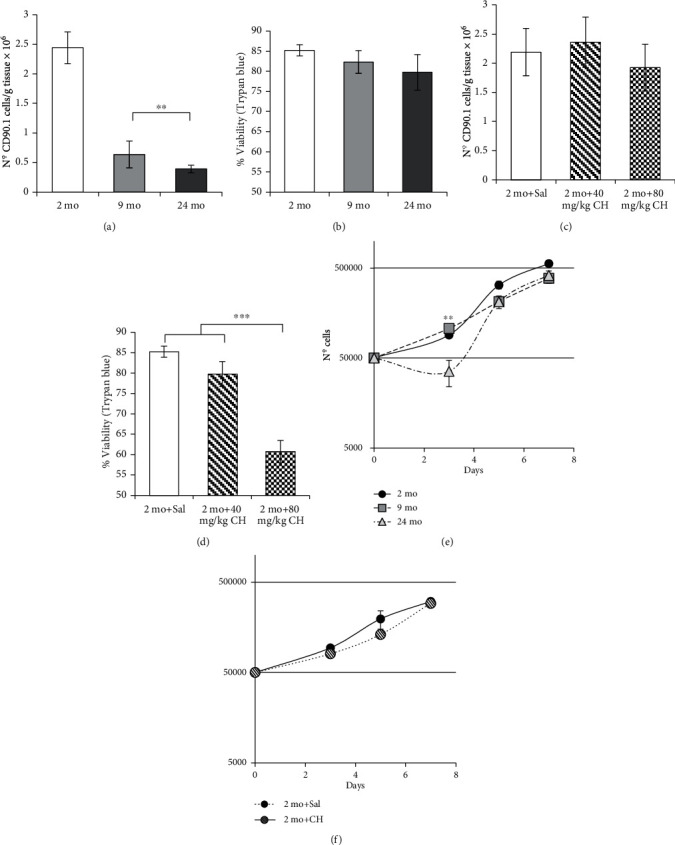
Effect of age and *in vivo* CH treatment on the viability, cell number, and cell growth in rat ADSCs. Quantitative score of CD90.1-positive cells per gram of tissue after immunomagnetic separation from 2- (*n* = 4), 9- (*n* = 4), and 24-month-old rats (*n* = 3) (a). Trypan blue exclusion test of cell viability in CD90.1-positive cells after immunomagnetic separation from 2- (*n* = 4), 9- (*n* = 4), and 24-month-old rats (*n* = 3) (b). Quantitative score of CD90.1-positive cells per gram of tissue after immunomagnetic separation from 2-month-old rats treated with saline (*n* = 4), 40 mg/kg/day of CH (*n* = 4), and 80 mg/kg/day of CH (*n* = 4) (c). Trypan blue exclusion test of cell viability in CD90.1-positive cells after immunomagnetic separation from 2-month-old rats treated with saline (*n* = 4), 40 mg/kg/day of CH (*n* = 5), and 80 mg/kg/day of CH (*n* = 5) (d). Cell growth curves of rADSCs from 2- (*n* = 3), 9- (*n* =  3), and 24-month-old rats (*n* = 4) (e). Cell growth curves of rADSCs from 2-month-old rats treated with saline (*n* = 4) and 40 mg/kg/day of CH (*n* = 4) (f). Values are the mean ± SEM. One-way ANOVA followed by Tukey's test was performed for multiple comparisons (^∗∗^*p* < 0.01 and ^∗∗∗^*p* < 0.001).

We found a significant decrease in the number of rADSCs in 9- and 24-month-old rats with respect to the young group (Figure 2(a)). On the contrary, we did not find significant differences in cell viability between these groups (Figure 2(b)).

In the case of the rats treated with 40 mg/kg/day, the number of rADSCs remained unaltered with no significant differences between the experimental groups (Figure 2(c)). However, when we measured the cell viability, the group treated with 80 mg/kg/day of CH showed a significant and important loss of viability (61 ± 2.71%) (Figure 2(d)).

The growth characteristics were assessed in rADSCs. The results showed that the proliferation of rADSCs from 24-month-old rats slows down during the lag phase (2-3 days) with respect to the 2- and 9-month-old groups (Figure 2(e)). These results indicate some kind of alteration in the cellular growth or adaptation to the culture environment of these cells. After a few days, the culture enters a period of most active growth similar to rADSCs obtained from younger animals. No change in the growth pattern of cultured cells was observed in rADSCs isolated from the group treated with 40 mg/kg/day of CH in comparison to the control group (Figure 2(f)). 80 mg/kg/day of the CH group was discarded for the determination of the growth curves because this concentration induced a significant decrease of cell viability in rADSCs (less than half of the cultures were viable).

### 3.4. Effect of Age and *In Vivo* CH Treatment on rADSCs' Pluripotency and Differentiation

**Figure 3:**
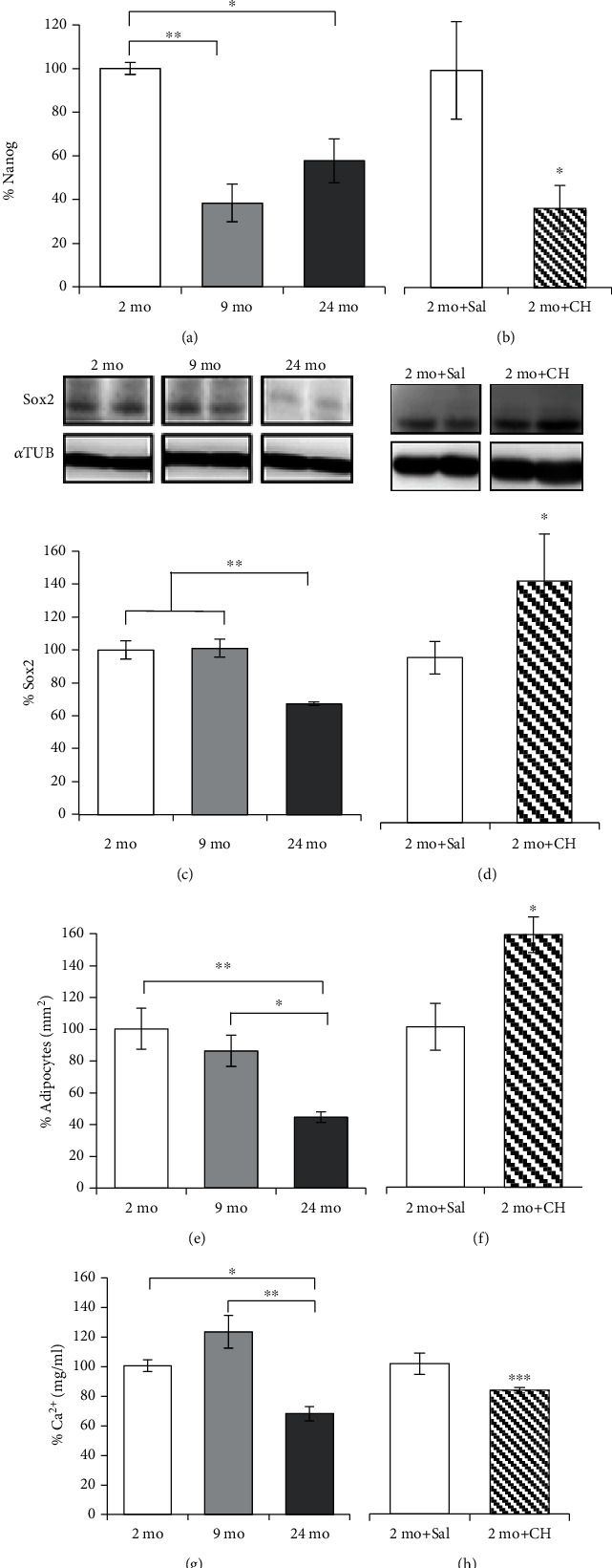
Effect of age and *in vivo* CH treatment on the levels of Nanog and Sox2 and differentiation potential in rADSCs. Levels of Nanog (a, b) and Sox2 (c, d) measured by WB in 2- (2 mo) ((a), *n* = 3; (c), *n* = 4), 9- (9 mo) (*n* = 4), and 24-month-old rats (24 mo) (*n* = 3) (a, c) and in 2-month-old rats treated with 40 mg/kg/day of CH (2 mo+CH) ((b), *n* = 5; (d), *n* = 4) or saline (2 mo+Sal) (*n* = 4) (b, d). Quantification of adipogenic differentiation by systematic counting of differentiated adipocytes (e, f) and osteogenic differentiation by spectrophotometry detection of Ca^2+^ (mg/ml) concentration after osteoblast formation (g, h) of rADSCs from 2- (2 mo) (*n* = 4), 9- (9 mo) (*n* = 4), and 24-month-old rats (24 mo) (*n* = 3) (e, g) and from 2-month-old rats treated with 40 mg/kg/day of CH (2 mo+CH) (*n* = 4) or saline (2 mo+Sal) ((f), *n* = 4; (h), *n* = 3) (f, h). Values are the mean (%) ± SEM expressed as percentages with respect to the value of the control group (100%). One-way ANOVA followed by Tukey's test was performed for multiple comparisons. ^∗^*p* < 0.05 and ^∗∗^*p* < 0.01 (a, c, e, and g). One-way ANOVA was performed for comparison of two groups. ^∗^*p* < 0.05 and ^∗∗∗^*p* < 0.001 (b, d, f, and h).

To determine whether age and *in vivo* CH treatment affect the pluripotency of rADSCs, we assessed the levels of Nanog and Sox2. In rADSCs, our results showed that Nanog levels significantly decreased up to 60% and 43% in 9- and 24-month-old rats, respectively, compared to the control group (Figure 3(a)). Similarly, the levels of Nanog in 2-month-old animals treated with 40 mg/kg/day of CH decreased up to 63.5% with respect to the control group (Figure 3(b)). The levels of Sox2 were only significantly lower (33%) in the 24-month-old group with respect to young animals (Figure 3(c)). While levels of Nanog decrease, Sox2 levels were upregulated (46%) in ADSCs obtained from CH-treated rats (Figure 3(d)).

We also quantified the capability of rADSCs obtained from rats of different ages and treated with CH to differentiate into adipocytes and osteoblasts when these cells are induced to do so *in vitro*. Our results showed that all samples (from 2-, 9-, and 24-month-old and CH-treated rats) were positive for adipogenic differentiation (Supplementary Figure 3A), as indicated by the presence of lipid vacuoles after staining with Oil Red O. Also, the percentage of adipocytes formed by differentiation of rADSCs decreased as a function of the age (Figure 3(e)). Thus, rADSCs from the 24-month-old group led to 65% fewer adipocytes than the 2-month-old rADSCs. On the contrary, we found a significant 39% increase in adipogenic differentiation in CH-treated rats compared to the control group (Figure 3(f)).

Regarding osteogenic differentiation, all samples were positive to staining with NBT/BCIP after the experimental induction, which indicates the presence of alkaline phosphatase (Supplementary Figure 3B). As to adipogenic differentiation, the osteogenic differentiation also decreased with age. Thus, the rADSCs obtained from the 24-month-old group had 32% less Ca^2+^ levels than the rADSCs obtained from the 2-month-old group (Figure 3(g)). The group treated with CH also showed a decrease of 38% in Ca^2+^ levels (Figure 3(h)). Therefore, osteogenic differentiation decreases with both age and CH treatment.

### 3.5. Effect of Age and *In Vivo* CH-Induced LP on eEF2, AMPK*α*, and SIRT1 Levels in rADSCs

Levels of different proteins have also been studied that can play a key role not only in the stem cell biology but also in any other cells: AMPK*α* and SIRT1. Because many biological actions of these two proteins converge on protein synthesis, specifically in eEF2, the levels of this protein were also included in this study.

**Figure 4:**
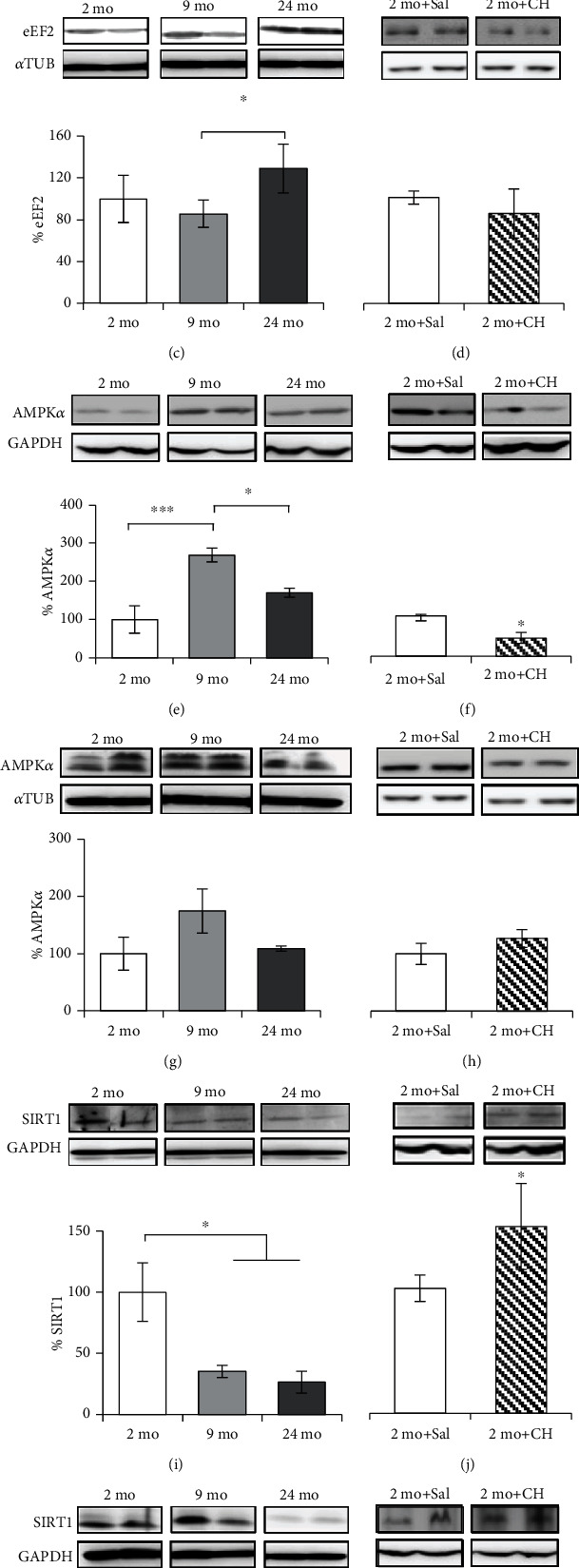
Quantification of eEF2, AMPK*α*, and SIRT1 in rADSCs. Optical densities of eEF2, AMPK*α*, and SIRT1 were measured by WB in the liver (a, b, e, f, i, and j) and rADSCs (c, d, g, h, k, and l) from 2- (2 mo, *n* = 3), 9- (9 mo, *n* = 4), and 24-month-old rats (24 mo, *n* = 3) (a, c, e, g, i, and k) and from 2-month-old rats treated with 40 mg/kg/day of CH (2 mo+CH, *n* = 4, except for (l), *n* = 5) or saline (2 mo+Sal, *n* = 4, except for (l), *n* = 3) (b, d, f, h, j, and l). Values are the mean (%) ± SEM expressed as percentages with respect to the value of the control group (100%). One-way ANOVA followed by Tukey's test was performed for multiple comparisons. ^∗^*p* < 0.05 and ^∗∗∗^*p* < 0.001 (a, b, e, f, i, and j). One-way ANOVA was performed for comparison of two groups. ^∗^*p* < 0.05 (c, d, k, and l).

In the rat liver, we observed a decrease in the level of eEF2 as a function of the age and after the CH treatment (Figures 4(a) and 4(b)). These findings agree with previous results reported from our laboratory [33–35, 50]. Thus, eEF2 levels were significantly reduced to 48 and 20% in 9- and 24-month-old rats, respectively, with respect to the young group (Figure 4(a)). CH also led to a significant decrease of 63% in the levels of eEF2 with respect to control values (Figure 4(b)). In rADSCs, eEF2 levels did not decrease with age but their levels increased by 44% in 24-month-old rats in comparison to the 9-month-old group (Figure 4(c)). However, CH treatment did not induce any change in the eEF2 levels (Figure 4(d)).

We have also found significant changes in the hepatic levels of AMPK during aging. There is a significant increase in the levels of this protein in 9-month-old rats with respect to young animals. Later, the results show a decrease of 66% in the levels of AMPK in the 24-month-old group with respect to 9-month-old rats (Figure 4(e)). These results agree with previous reports indicating that AMPK decreases with age [29, 51, 52]. Similar to aging, CH treatment decreased the hepatic levels of AMPK by 55% (Figure 4(f)). In rADSCs, the changes in the expression of AMPK with age were similar to those found in the liver but no significant reduction was observed in CH-treated rats (Figures 4(g) and 4(h)).

The levels of SIRT1 were also studied in both the rat liver and the rADSCs. In hepatic cells, our results showed a significant decrease of 65% between the 2-month-old and 9-month-old groups, with no further decrease in the 24-month-old group (Figure 4(i)). On the contrary, CH treatment led to an increase in the levels of SIRT1 (Figure 4(j)). In rADSCs, the levels of SIRT1 remain unchanged in 9-month-old rats and rise to 86% in 24-month-old groups, respectively, with respect to the young group (Figure 4(k)). As in the liver, CH treatment increased the levels of SIRT1 by 46% with respect to the control group (Figure 4(l)).

### 3.6. Effect of Age in hADSCs

**Figure 5:**
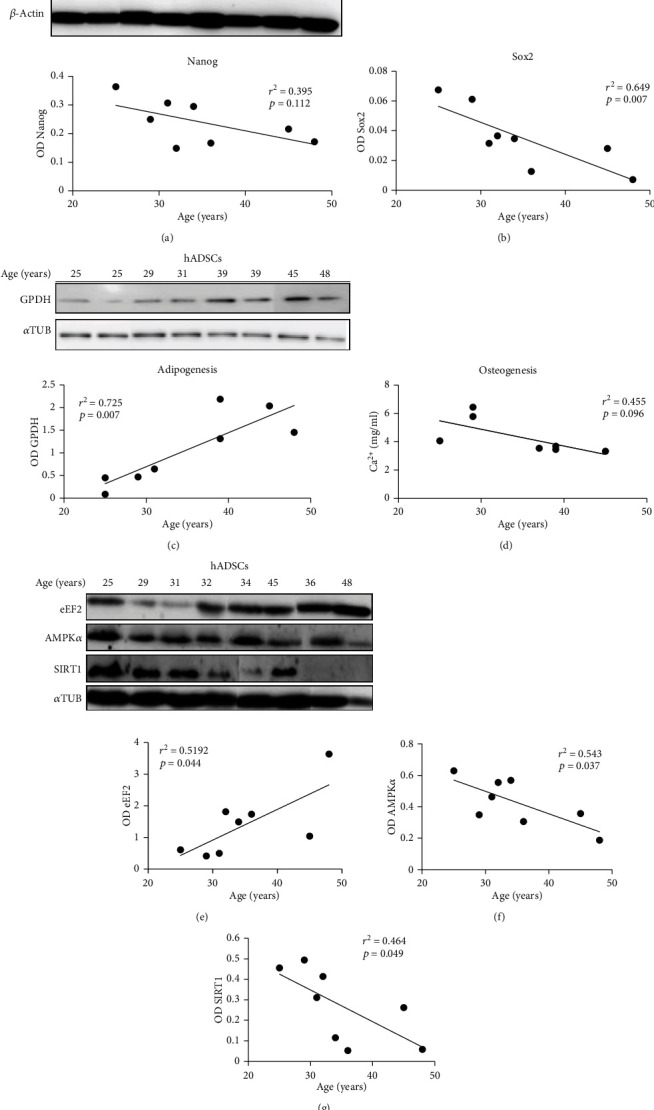
Effect of age in hADSCs. The pluripotency factors Nanog (a) and Sox2 (b) were measured in hADSCs by WB as well as the levels of GPDH to determine adipogenesis (c). For osteogenesis assessment, the levels of Ca^2+^ (mg/ml) was measured by spectrophotometry at 610 nm (d). Optical densities of eEF2 (e), AMPK*α* (f), and SIRT1 (g) were measured by WB. Results are expressed as optical density (OD) after normalization with loading control protein OD values. Correlation analysis (Pearson's *r* test) was performed to measure the association between the two variables.

hADSCs from patients of different ages (25 to 48 years old) were assessed to determine both their levels of Nanog and Sox2, differentiation capabilities into adipocytes and osteoblasts, and protein levels of eEF2, AMPK*α*, and SIRT1. Our results showed that only Sox2 decreased significantly with age and no significant change was found for Nanog (Figures 5(a) and 5(b)). In order to make a correct comparison between humans and rats, we should have had a wider range of human ages to include younger and older individuals. However, if one considers only the comparison between middle-aged and older people, it is tempting to affirm that results are somehow similar. In rats, the higher values of Nanog were found only in young individuals with no significant differences between middle-aged and aged animals (Figure 3(a)); Sox2 significantly decreased only in very old individuals (Figure 3(c)).

In regard to the differentiation potential of hADSCs, the results were different from those found in rADSCs. Thus, adipogenic differentiation increased significantly with age (Figure 5(c)), and no change in the capacity of differentiation into osteoblasts was observed (Figure 5(d)). All hADSC samples tested showed adipocyte and osteoblast differentiation capacity (Supplementary Figure 4).

Finally, we assessed the levels of eEF2 (Figure 5(e)), AMPK*α* (Figure 5(f)), and SIRT1 (Figure 5(g)) in hADSCs obtained from patients of different ages. As can be seen, eEF2 levels have a significant positive correlation with age; meanwhile, AMPK*α* and SIRT1 tend to decrease during aging.

### 3.7. *In Vitro* Effect of CH on Human and Rat ADSC Viability

**Figure 6:**
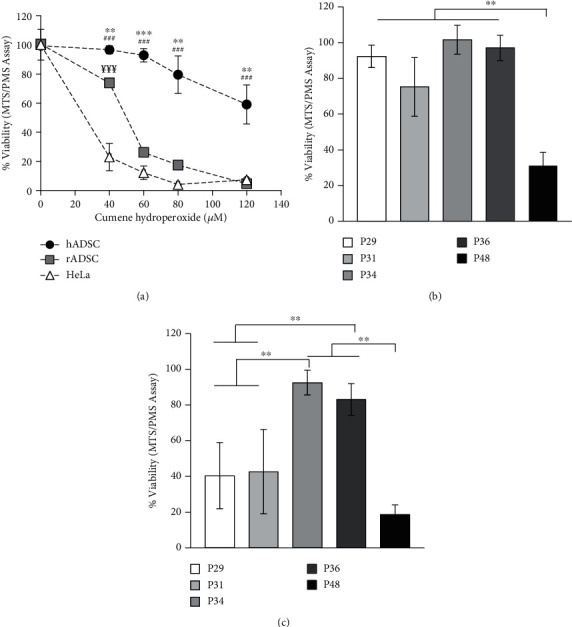
Effect of age and *in vitro* CH treatment on human and rat ADSC viability. *In vitro* MTS/PMS viability assessment of the hADSCs, rADSCs, and HeLa cell line (as a human nonstem cell control) under different concentrations of CH (0-120 *μ*M) (*n* = 5) (a). *In vitro* MTS/PMS viability assessment of hADSCs from patients of different ages (29-48 years old) treated with 80 *μ*M (b) and 120 *μ*M (c) of CH (*n* = 8 technical replicates from each age). Statistical analysis: values are the mean ± SEM. One-way ANOVA followed by Tukey's test was performed for multiple comparisons. ^∗∗^*p* < 0.01 and ^∗∗∗^*p* < 0.001 (hADSCs vs. HeLa); ^##^*p* < 0.01 and ^###^*p* < 0.001 (hADSCs vs. rADSCs); and ^¥¥¥^*p* < 0.001 (rADSCs vs. HeLa) (a). ^∗∗^*p* < 0.01 (between ages).

We also studied the possible difference in the vulnerability of rADSCs and hADSCs under the oxidative stress caused by CH. Using MTS/PMS assay, we determined the *in vitro* viability of the rat ADSCs (9-month-old), human (25-48 years old) ADSCs, and HeLa cell line (as a human nonstem cell control) exposed to different concentrations of CH (0-120 *μ*M). The results show that hADSCs were the most resistant to CH, especially at high doses (Figure 6(a)). At a low dose of CH (40 *μ*M), both hADSCs and rADSCs were more resistant than HeLa cells. At higher doses, there were no differences in viability between rADSCs and HeLa cells; meanwhile, hADSCs kept high levels of viability (59.0 ± 13.4%).

The effect of CH was also tested *in vitro* on hADSCs from patients of different ages. Cells were treated with two concentrations of the oxidant, 80 and 120 *μ*M for 3 h. hADSCs from P48 (48 years old) was the most sensitive sample up to 80 *μ*M of CH (cell viability of 31.12 ± 7.49%). The viabilities of the other samples were similar and remain unaltered (Figure 6(b)). However, when CH concentration increased up to 120 *μ*M, the viabilities of P29 (29 years old) and P31 (31 years old) were significantly affected, to reach a similar value to that found in P48 (18.76 ± 5.37%) (Figure 6(c)).

## 4. Discussion

In this study, we investigated several aspects of stem cell biology that are related to their capacity to proliferate, differentiate, and resist oxidative stress. We have focused on different molecular pathways that directly and indirectly drive these biological aspects and how they can be affected by aging and oxidative stress. The effect of aging was addressed because an important issue in autologous SC transplant is whether stem cell-based therapies should be restricted to certain donor age. The successful clinical application of cell therapy depends on the ability of stem cells to replace cells in damaged tissues, where stem cells might have to adapt to an adverse environment, for instance, oxidative stress. High oxidative stress contributes to general biomolecular damages resulting in an impairment of physiological functions that takes place in both aging and chronic age-related diseases. Moreover, uncontrolled oxidative stress is a hallmark of the aging process which is associated with mitochondrial dysfunction, inhibition of autophagy, and accelerated aging [53, 54]. Oxidative stress has also been associated with cellular senescence, persistent inflammation, telomere erosion, and dysregulation of intracellular metabolites that promote apoptosis [55]. Given that stem cells need to adapt to an oxidative stress adverse environment and that impairment of stem cell regenerative capacity is associated with accelerated aging by decreasing proliferation and increasing senescence and apoptosis, here we discussed how aging and oxidative stress induced by CH can affect the ADSCs' therapeutic capacity and/or their survival capabilities in the site of transplantation.

The present study has been carried out in ADSCs obtained from humans and rats of different ages. ADSCs were obtained from SVF of lipoaspirates digested enzymatically followed by immunomagnetic isolation with antibodies conjugated to microbeads [39, 40, 49, 56]. This isolation method allows us to obtain a more homogeneous MSC population (Supplementary Figures 1B and 2B) than other methods based on SVF seeding [41, 57]. This is because SVF is like a soup that contains different cell populations with several types of cells with multipotent capacity [2]. In the human bone marrow, the percentage of MSC CD271^+^ is around 1.16% of total SVF cell populations [58]. In lipoaspirates, the percentage can vary. On average, starting from 300 ml of lipoaspirate, we obtained 1 × 10^7^ cells in the SVF and only around 0.75‐1.25 × 10^5^ CD271^+^ cells (0.75-1.25%) were seeded onto T25 flasks. In the case of rats, the percentage of CD90.1^+^ cells obtained in SVF was a bit higher (2-3%). We cannot ensure that these differences are related to different species or to the fact that we used different markers for immunomagnetic separation.

To understand the influence of oxidative stress on MSCs, we performed both *in vivo* (rADSCs) and *in vitro* (hADSCs) studies. We used CH as an experimental model of oxidative stress, which increases lipid peroxidation when it is injected *in vivo* and *ex vivo* or added to cell culture [11, 33–35, 59]. It has been reported that 4-HNE levels increase with age and after CH treatment both *in vitro* and *in vivo* in a dose-dependent manner in the liver [33]. The measurement of hepatic 4-HNE is used in the present study as a positive control of the CH treatment. We observe that 40 mg/kg/day treatment produced a similar increase in the levels of 4-HNE-protein adducts in both the stem and hepatic rat cells (Figure 1). This result suggests that rADSCs are affected by oxidative stress, in this case by lipid peroxidation, like any other somatic cells, probably because the mechanisms involved in preventing ROS damage are similar.

Oxidative stress affected not only the cellular viability but also the number of MSCs present in adipose tissue. It has been described that the number of cells and the proliferative and differentiation capacities of ADSCs decrease with age in different species [60–62]. In our case, there is a considerable decrease in the number of rADSCs with age (Figure 2(a)), which is evident in adult age. However, viability is not affected (Figure 2(b)). This indicates that a lower number of MSCs are present in old adipose tissue but the viability of the remaining cells is similar. According to this, and in order to regenerate tissue with cell therapy, a higher amount of adipose tissue should be used to get a high number of cells if the donor is older.

Contrary to what is observed in aging, *in vivo* treatment with CH did not affect the number of cells per gram of adipose tissue (Figure 2(c)), but it did affect the viability (Figure 2(d)). We can speculate on the possibility that ADSCs from young animals are capable of rapidly replacing the stem cells that are lost either by the direct action of CH or by the oxidative stress-induced differentiation. Under these circumstances, the ADSC pool is composed of newly formed cells and CH-affected cells whose viability has been affected but not eliminated by necrosis or apoptosis. The observed result with altered viability but not the number of cells may be due to the fact that the separation system used in this work (immunomagnetic isolation of CD90.1-positive cells) does not discriminate between these two subpopulations.

The lack of concordance between the effect of age and CH *in vivo* treatment on the number and viability of the cells may be related to the exposure time to oxidative stress. In the case of treatment with CH, oxidative stress exposure can be considered short to affect the number of stem cells in relation to the persistent/continuous exposure to oxidative stress over the life span. This points in the direction that it would be convenient to assess the general status of oxidative stress of the donor before a cell therapy strategy is being considered. Also, a higher amount of adipose tissue should be used to get a higher number of cells if the donor is older or cells are isolated from an oxidative stress environment before a cell therapy strategy is being considered.

It is also important to highlight the results shown in Figure 2(e) on the rADSC growth curves once they are cultured. The results show that older donor cells seem to have more difficulty adapting to the culture conditions, but finally, they end up growing at the same speed as the rADSCs from younger donors. This result suggests that it will take longer to get the therapeutic effects when using ADSCs from older donors.

In this study, we also investigated the age-related changes in some molecules responsible for SC proliferation and differentiation. Within this group, the transcription factors Nanog and Sox2 are crucial for the efficient maintenance of pluripotent cell identity [23, 63, 64]. We hypothesize that both aging and CH treatment can modify the expression of pluripotency factors and that could have consequences in the cell fate differentiation. In hADSCs, our results show that the expression of Nanog remains unchanged along with the age of the donors but the Sox2 level decreased significantly (Figures 5(a) and 5(b)). In rats, the same change was observed in Sox2 expression but Nanog levels were also affected during aging (Figure 3). In principle, these results may appear different with respect to human cells. However, it is necessary to bear in mind that the age range used in the human study does not include individuals as young or as old as in the group of rats, where no additional changes are observed in the expression of Nanog after 9 months (Figure 3(a)). These results agree with a previous report showing that in human bone marrow-derived mesenchymal stromal cells, Nanog expression correlation with age was not statistically significant either [16]. That supports the idea that Nanog is only upregulated in young cells, where it not only can be considered a youth indicator but also can reverse the senescence in stem cells [22, 65]. To confirm this, more studies are necessary with hADSCs from younger patients.

In addition to the study of the levels of these relevant pluripotency transcription factors, we also evaluated the effect of aging on human ADSCs' potentiality to differentiate into adipocytes and osteoblasts. The results show that the potentiality to differentiate into adipocytes is promoted (Figure 5(c)). These results show some similarities between the effect of aging in humans and the effect of CH in rats (Figure 3(f)) since both situations increase the adipogenic differentiation. In addition, oxidative stress also affected osteogenesis. Taken together, these results suggest that oxidative stress could be responsible for the changes in the differentiation capacity observed in aging, which would lead to a decrease in the pool of MSCs available for regenerative purposes. Obviously, the pathways that regulate differentiation are complex [66] and, probably, are specific to each species and are modulated by several metabolic conditions. Thus, in rats, both Sox2 and Nanog expression decreased with age, concomitantly to the decline of osteogenic and adipogenic differentiation. In any case, special care must be taken when trying to extrapolate results from murine models to humans. However, we cannot forget that those results should be interpreted having in mind the limitation of this study regarding the sampling number and the range of age in hADSCs.

When rats were treated with CH, the levels of Nanog decreased but those of Sox2 increased (Figure 3). These changes were concomitant to both the increase in the potentiality to differentiating into adipocytes and the decline in osteoblasts. This agrees with previous reports where the increase of Sox2 induces or stimulates adipogenic differentiation when Nanog levels are low, which is necessary for stem cell differentiation [23, 67]. Park et al. (2012) observed that oxidative stress increased the levels of Sox2 and Oct4, but they did not mention any reference to Nanog. Ectopic expression of Nanog maintained the stemness state [22, 65] and inhibited adipogenesis [68]. The increment of Sox2 by CH could be related to high levels of SIRT1 (Figure 4(l)). By promoting Sox2 deacetylation, SIRT1 would avoid its degradation mediated by the proteasome [69, 70]. This increment poses a problem in cell differentiation, because the more the pluripotent capacity, the more sensitive it would have to be to differentiate into adipocytes [23, 69, 70]. That suggests that young rADSCs from animals treated with CH *in vivo* tend to differentiate into adipocytes; meanwhile, older ones could not do it because of the low Nanog and Sox2 levels. However, other authors suggest that cells with high levels of Sox2 inhibit both adipogenic and osteogenic differentiation [71]. We suggest that Sox2 inhibits both adipogenic and osteogenic differentiation in the presence of Nanog [63, 67]. Nanog maintains the self-renewal state through Oct4-Sox2-Nanog transcription factor regulation [63] and also under certain conditions such as lipid peroxidation-mediated caspase-3 activation [72], which would promote Nanog destabilization [73], destroying the self-renewal state and leaving Sox2 free to induce the differentiation process.

In addition to studying aspects related to the mechanisms of pluripotency and differentiation of the ADSCs, in this work, we have determined levels of other proteins that can play an important role in their cell biology and aging. These proteins are AMPK, SIRT1, and eEF2, which are widely regarded as critical regulators of a wide variety of cellular processes.

AMPK is a sensor of cellular energy status, a major inducer of autophagy and stress response [26]. It has been described that transitory AMPK activation would promote cell survival pathways preferentially, whereas sustained AMPK activation would trigger cell death [74]. AMPK also plays an important role in MSC differentiation, promoting osteogenic differentiation and suppressing adipogenic differentiation [69].

In hADSCs, our results show a significant decrease in levels of AMPK with age (Figure 5(f)). This decrease should be considered together with the changes in eEF2. In order to keep an active protein synthesis under a specific metabolic situation, the AMPK activity must be reduced. However, low levels of AMPK in ADSCs during aging could affect its homeostasis and the ability to respond to oxidative stress injury, not only in their original niche but also after they have been transplanted into the injured site. In addition, considering the role of this enzyme in the differentiation of stem cells, the decrease in AMPK levels with age may contribute to the increased potentiality to differentiate into the adipocytes observed with age [75, 76].

A similar decline was observed in the expression of AMPK in rADSCs from ages 9 to 24 months (Figure 4(g)). The same changes were observed in the liver (Figure 4(e)). Recent studies have shown that AMPK activation, in response to various stimuli, such as exercise and muscle contraction, gradually declines during aging [77]. Although mechanisms underlying this have not been elucidated, it is possible that the age-related increased chronic inflammation levels suppress AMPK activation in aged tissues [78].

It is noteworthy that CH treatment only affected the expression of hepatic AMPK but not in rADSCs. This result may be related to what has previously been mentioned: the effects of oxidative stress depend on whether it is transient or more permanent. Also, it may be related to the functions of the AMPK being tissue-specific as has been previously described [79].

In regard to the levels of eEF2, our results show that aging and oxidative stress decrease the levels of eEF2 in the liver. This most likely affects the global protein synthesis in that tissue. However, eEF2 levels of both rADSCs and hADSCs increase with age (Figures 4(c) and 5(e)). It is possible that eEF2 is protected in some way so that protein synthesis in ADSCs can take place under conditions where optimal functions are compromised, such as oxidative damage, and thus contribute to tissue repair. These results show once more the different biology of SCs in comparison to other cells since these cells must work under harmful conditions, which is common in damaged tissues.

Protein synthesis is a process that consumes a large amount of energy and therefore is inhibited in the conditions where energy is limited to conserve cellular ATP so that the ATP is used for repair and recycling of cellular components. AMPK inhibits cap-dependent translation during both the initiation and elongation steps by indirectly inhibiting mTORC1. Furthermore, AMPK directly inhibits translational elongation by phosphorylating and activating eEF2K, which phosphorylates and inhibits eEF2 [80]. However, the expression of those genes involved in cell repair and survival is also required during energy stress [81]. Interestingly, AMPK can perform these functions by switching translation from cap-dependent to cap-independent mechanisms; thus, global synthesis is inhibited but IRES-mediated synthesis is activated [82]. This type of synthesis is less dependent on an initiator factor and is activated under cellular stress conditions. eEF2 participates actively in both global and IRES-mediated synthesis. In order to participate in one or the other, it is necessary that certain modifications occur in its molecule. Maybe this is the reason why several regulatory mechanisms converge in their molecule. So, it makes sense that the levels of AMPK and eEF2 are opposite under conditions where cellular biology is challenged, as we show in ADSCs of different ages in rats and humans. Some reports have shown that MSCs synthesize fewer amounts of proteins than other cells because they are quiescent most of the time [83]. Supposedly, MSCs should be more active during aging where errors and molecular damages accumulate. In this hypothetical situation and in the case MSCs are not affected, their protein synthesis machinery should be activated to carry out the maintenance, repair, or differentiation process. According to this, our results show that old organisms have higher levels of eEF2. However, the final restorative effect cannot be correctly performed probably due to the decline of pluripotency factors. The fact that these results were not seen in rats treated with CH can perhaps be due to the treatment no longer being enough or to the fact that both proteins have different specific protection mechanisms providing these cells with the ability to work under adverse conditions.

The levels of SIRT1 were also studied in this work. SIRT1 is another class of protective enzymes that play an important role in cellular metabolism, health, and aging. Although it is clear that SIRT1 is modulated by oxidative stress, the molecular mechanisms are not well understood [84, 85]. It has been suggested that SIRT1 deacetylates FOXO1 and FOXO3 and increases the cellular resistance to oxidative stress [86]. Also, in adult mouse hearts, SIRT1 was significantly upregulated (4-fold) in response to oxidative stress (paraquat injection). Similarly, a 3-fold increase in SIRT1 levels was observed in old versus young monkey hearts [87]. Our results agree with these studies, because CH treatment increased the levels of SIRT1 in the liver and rADSCs. However, it has been proposed that oxidative stress induces SIRT1 expression as a compensatory mechanism, while harsh or prolonged oxidant conditions result in dysfunctional modified SIRT1 more prone to degradation by the proteasome. This could explain why levels of SIRT1 in rADSCs and hADSCs decrease with age (Figures 4(i) and 5(g)). In addition, SIRT1 plays an important role in MSC differentiation. SIRT1 is negatively correlated with adipogenesis and reduction of bone mass so that SIRT1 favors MSC differentiation toward osteoblast lineage at the expense of adipocyte lineage [69, 88]. Therefore, the decrease in SIRT1, along with the decrease in AMPK levels observed with age, may contribute to the increase in ADSCs' potentiality to differentiate into adipocytes observed in hADSCs with age in our study.

The results also show a different vulnerability of rADSCs and hADSCs when subjected to oxidative stress response *in vitro*. Comparing the cellular viability after CH treatment, we observed that hADSCs are more resistant than those isolated from rats and other cell types such as HeLa (Figure 6(a)). This result agrees with other authors' results [89–91]. It makes sense that ADSCs' behavior is different from other somatic cells so that they can resist harmful events and induce their regenerative functions, at least within certain limits that do not affect their own survival. Further studies would be necessary to investigate whether ADSCs have extra resistance to cope with oxidative stress and/or high levels of 4-HNE in relation to other somatic cells. Another important aspect is that hADSCs are more resistant than rADSCs. It could be possible that hADSCs have higher biochemical robustness than ADSCs from other species with shorter life span potential as it has been described by Cutler [92]. When we measure the cellular viability in hADSCs of different ages, the results show that the viability is sensitive to changes in the oxidative environment (Figures 6(b) and 6(c)). These results suggested that not all hADSCs had the same resistance to lipid peroxidation. These results must be considered preliminary due to the sampling number limitation. Further studies are required to profound in the correlation between age and oxidative resistance. Nonetheless, this result anticipates that an antioxidant treatment should be considered a potential coadjutant treatment to cell-based therapies.

Our findings conclude that before performing a cell therapy with ADSCs, several biochemical parameters should be assessed on these cells. Not only the number of cells and viability are important but also other determinations such as oxidative stress status or differentiation capacity of the cells should be assessed. This would allow more information on the biochemical robustness of the injected cells and help to predict the therapeutic result, especially in elder patients, where probably ADSCs do not carry out their biological functions in an optimal way.

## Figures and Tables

**Table 1:** Basal patient characteristics.

Patients included	*n* = 12
Age, mean ± SEM	34.3 ± 2.1 (25, 25, 29, 29, 31, 32, 34, 36, 39, 39, 45, and 48 years)
Surgery type	Abdominoplasty
BMI (kg/m^2^)	24-29

## Data Availability

The data that support the findings of this study are available from the corresponding author upon reasonable request.

## Abbreviations

hADSCs: Human adipose-derived stem cells
rADSCs: Rat adipose-derived stem cells
SVF: Stromal vascular fraction
MSCs: Mesenchymal stem/stromal cells
SCs: Stem cells.

## Supplementary Materials

Supplementary Materials Supplementary Figure 1: rat adipose-derived stem cell characterization. Cell culture images of adherent rat ADSCs and obtained after immunomagnetic isolation using CD90.1 (A). Flow cytometry analysis expressed a high percentage of CD29/CD90.1 markers (98.0 ± 0.9%) and low expression of CD11b/CD34/CD45 markers (<3%) in rADSCs (B). rADSCs were differentiated into adipocytes (Oil Red O staining), osteoblasts (alkaline phosphatase staining), and chondroblasts (immunocytofluorescence of anti-aggrecan) (C). Scale bar = 50 *μ*m. Supplementary Figure 2: human adipose-derived stem cell characterization. Cell culture images of adherent human ADSCs obtained after immunomagnetic isolation using CD271 (A). Flow cytometry analysis expressed a high percentage of CD90/CD105 (99.67 ± 0.32%) and CD90/CD73 (99.69 ± 0.2%) markers and low expression of CD14/CD20/CD34/CD45 (<0.5%) in hADSCs (B). hADSCs obtained from patients of different ages (25 and 48 years old) were differentiated into osteoblasts and adipocytes, which were detected by alkaline phosphatase and Oil Red O staining, respectively (C). Scale bar = 50 *μ*m. Supplementary Figure 3: rat adipose-derived stem cell adipogenic/osteogenic differentiation. Images from adipocytes pre- and poststained with Oil Red O (A) and osteoblasts stained with NBT/BCIP substrate (B) after differentiation induction of ADSCs from 2-, 9-, and 24-month-old rats and from 2-month-old rats treated with 40 mg/kg/day of CH or saline. Scale bar = 50 *μ*m. Supplementary Figure 4: human adipose-derived stem cell adipogenic/osteogenic differentiation. hADSCs obtained from patients of different ages (25-48 years old) were differentiated into osteoblasts and adipocytes, which were detected by alkaline phosphatase or visual vacuole confirmation (sometimes with Oil Red O staining, depending on the availability of the cells to perform all experiments). Note that not all samples were stained with Oil Red O dye because those implied to perform technical duplicates, which sometimes were impossible to the low number of cells available to perform all the experiments. Scale bar = 50 *μ*m.
