# Supplementary figures and images for: Effect of Age and Lipoperoxidation in Rat and Human Adipose Tissue-Derived Stem Cells
Source: Oxid Med Cell Longev. 2020 Dec 9;2020:6473279. doi: 10.1155/2020/6473279 (PMC7775166; doi:10.1155/2020/6473279)

**A**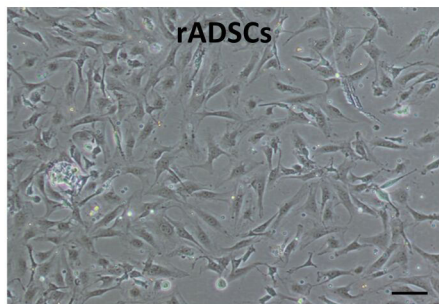**B**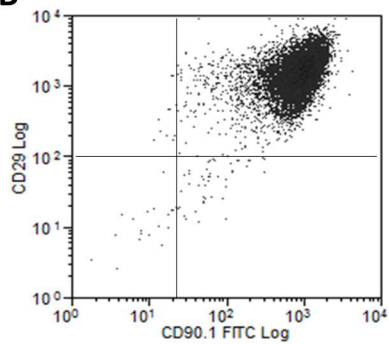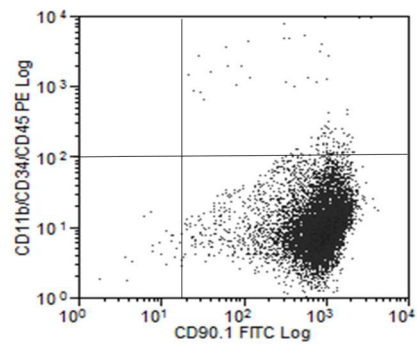**C****Adipocytes**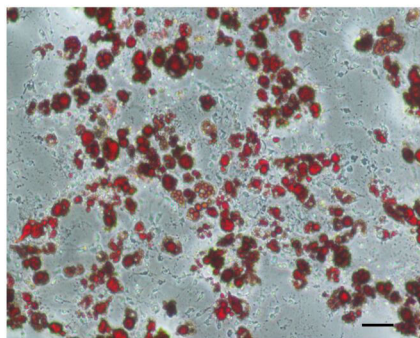**Osteoblasts**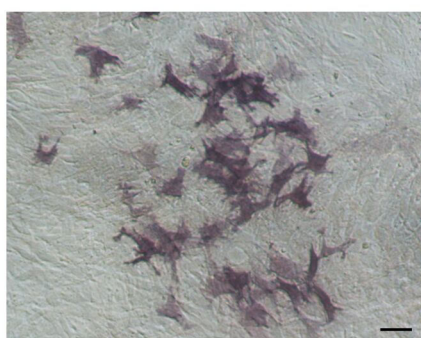**Chondroblasts**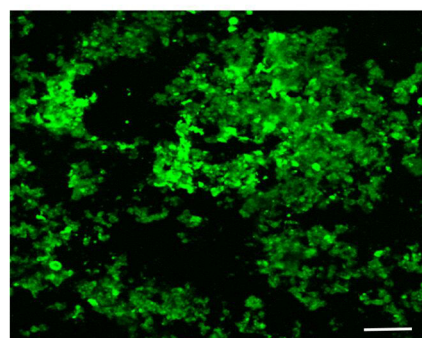

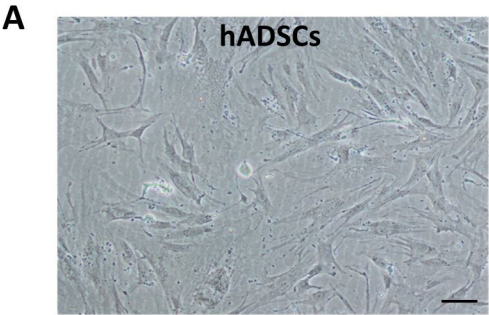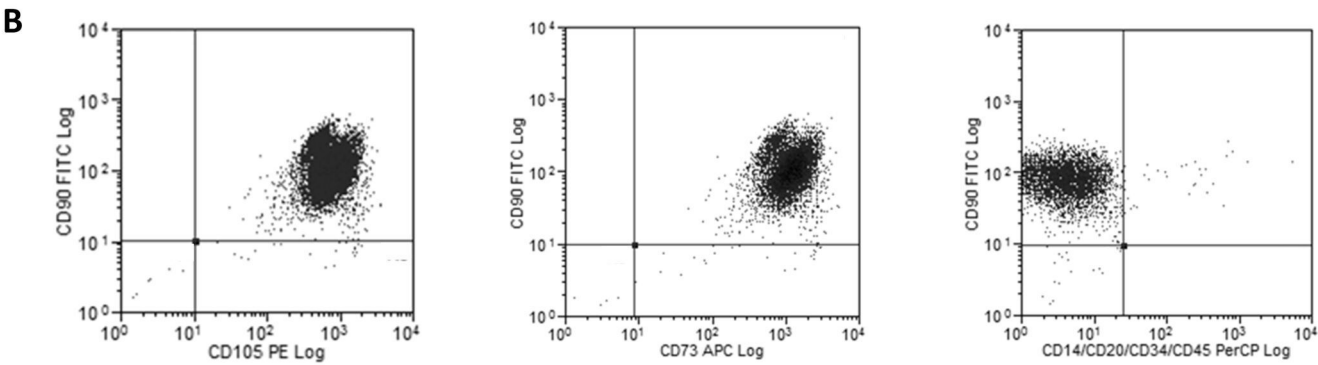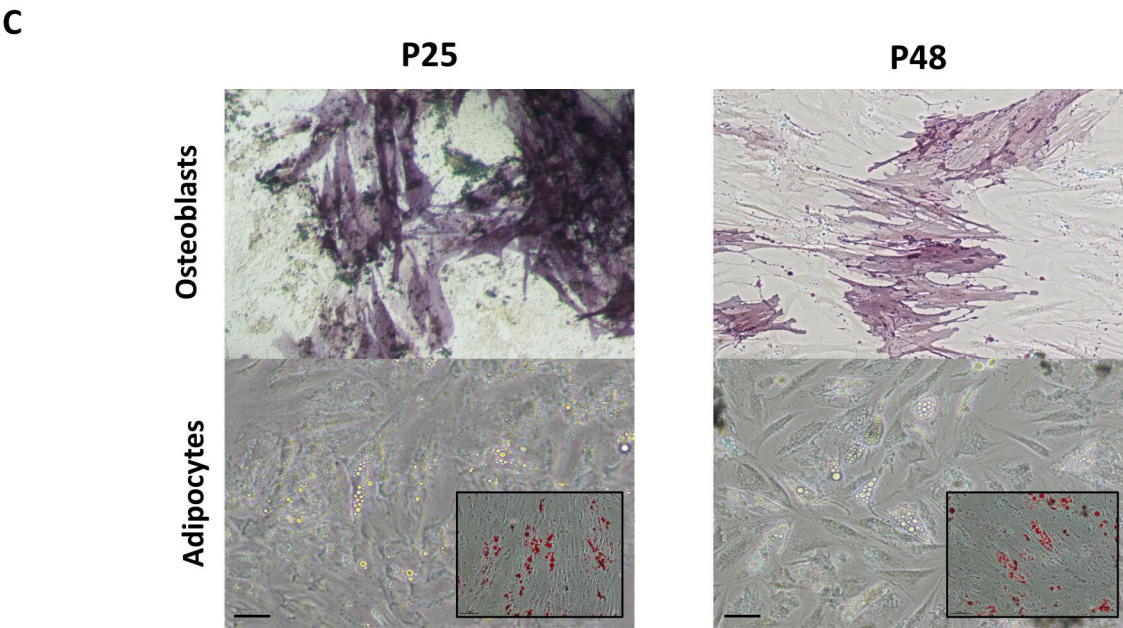

**Suppl Figure 2**

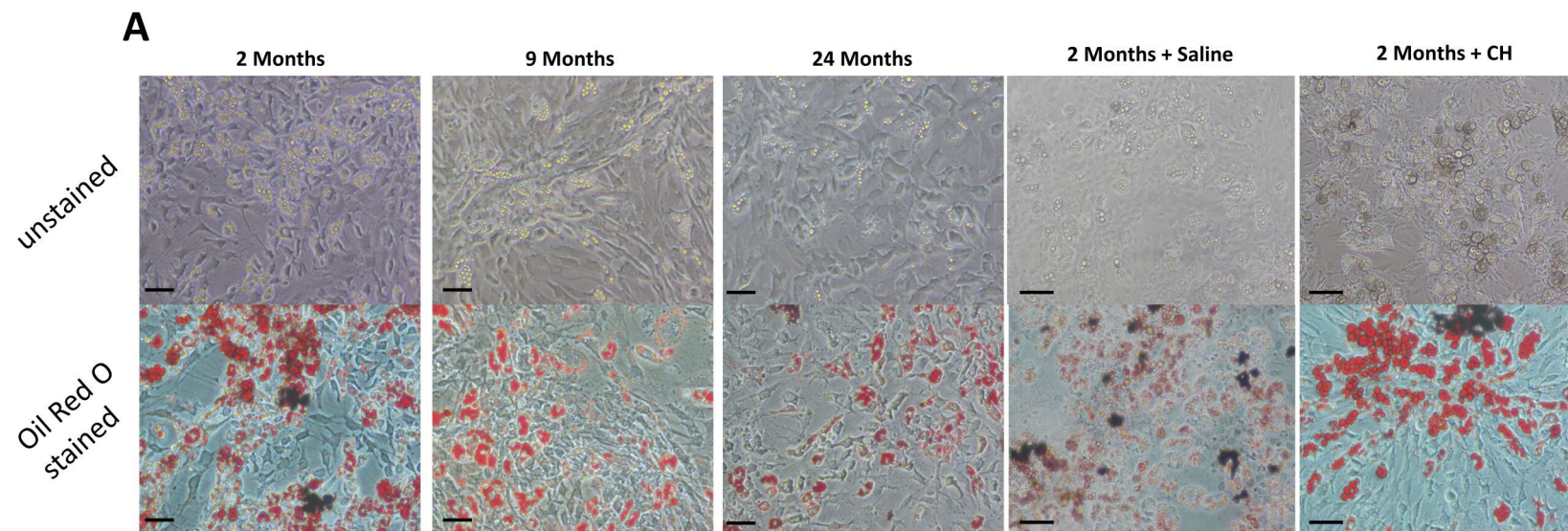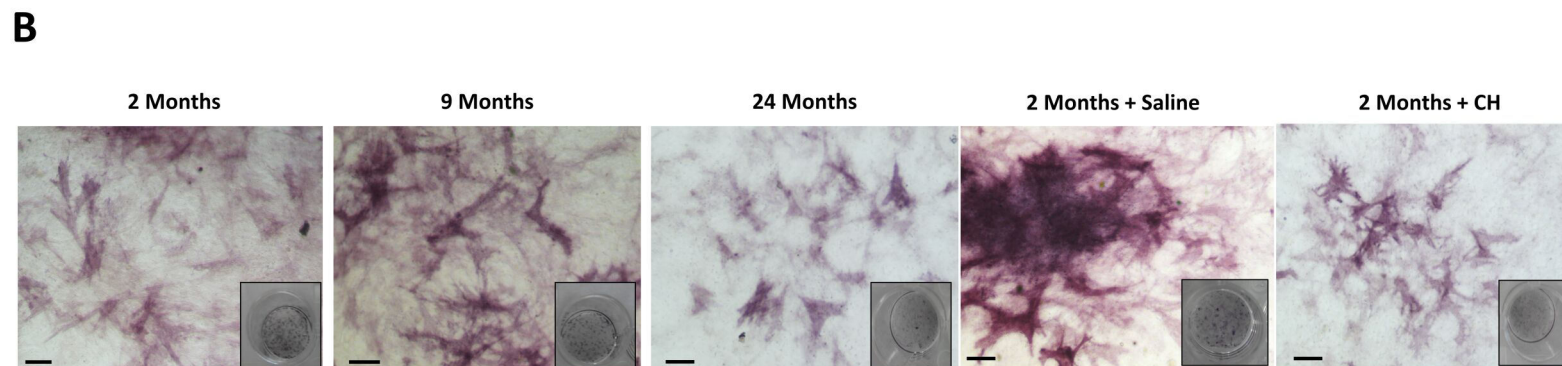

**Supplementary Figure 3**

**P25**

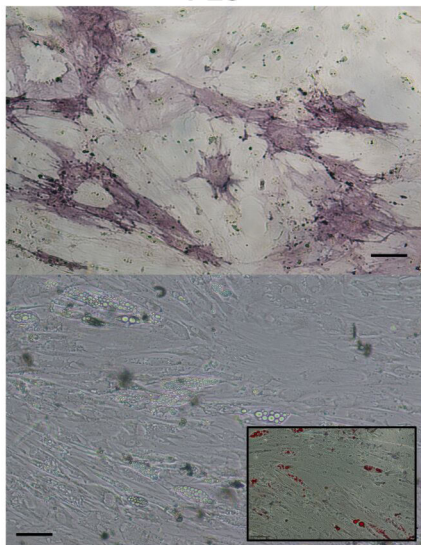

**P29**

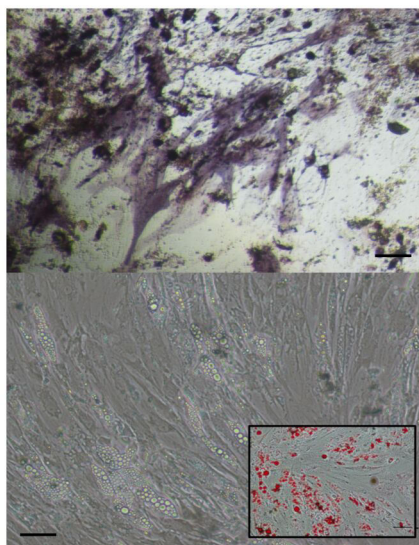

**P31**

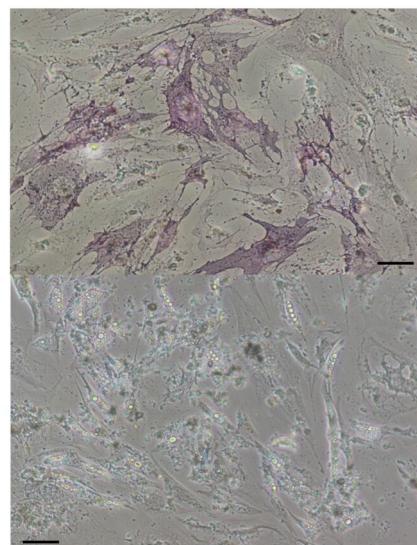

**P34**

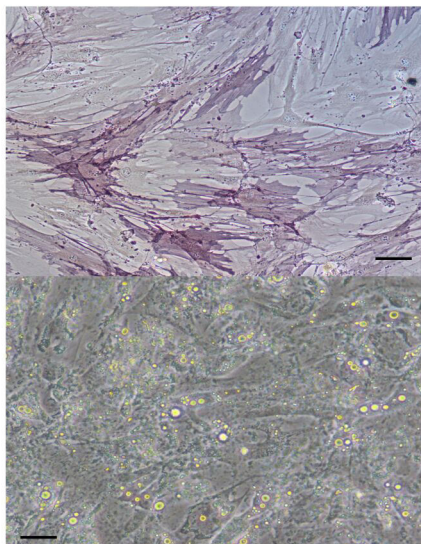

**P36**

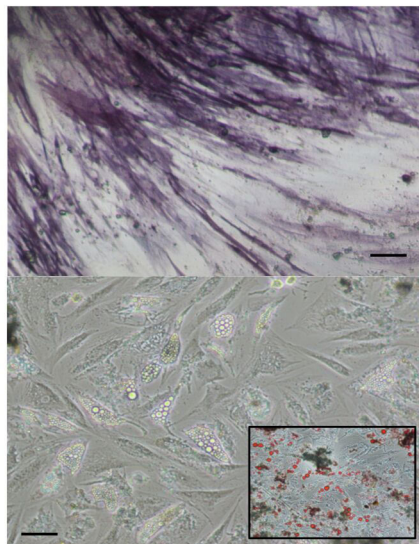

**P39**

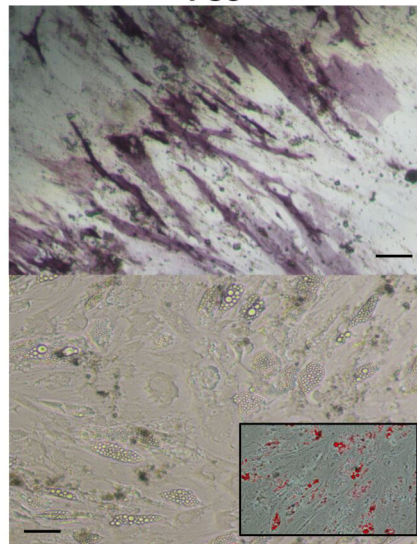

**P45**

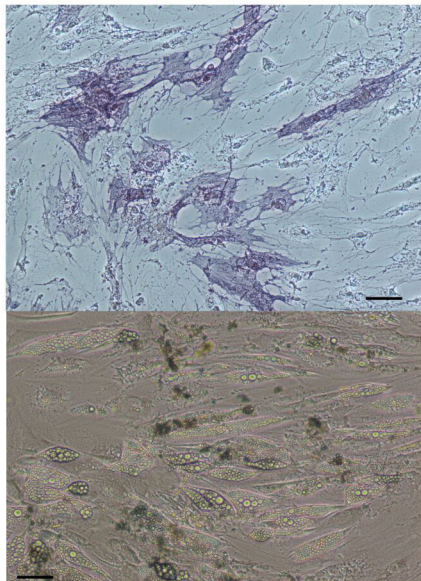

**P48**

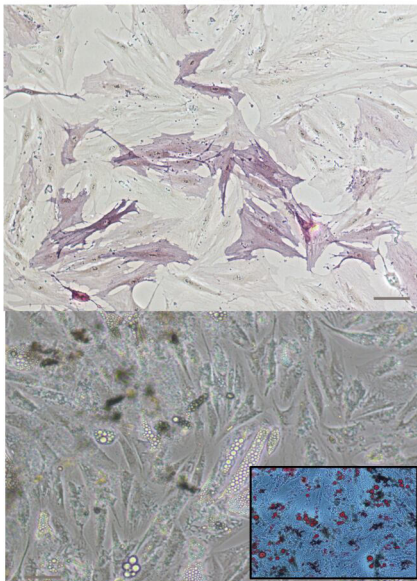

Supplement: Supplementary Materials — Supplementary Figure 1: rat adipose-derived stem cell characterization. Cell culture images of adherent rat ADSCs and obtained after immunomagnetic isolation using CD90.1 (A). Flow cytometry analysis expressed a high percentage of CD29/CD90.1 markers (98.0 ± 0.9%) and low expression of CD11b/CD34/CD45 markers (<3%) in rADSCs (B). rADSCs were differentiated into adipocytes (Oil Red O staining), osteoblasts (alkaline phosphatase staining), and chondroblasts (immunocytofluorescence of anti-aggrecan) (C). Scale bar = 50 μm. Supplementary Figure 2: human adipose-derived stem cell characterization. Cell culture images of adherent human ADSCs obtained after immunomagnetic isolation using CD271 (A). Flow cytometry analysis expressed a high percentage of CD90/CD105 (99.67 ± 0.32%) and CD90/CD73 (99.69 ± 0.2%) markers and low expression of CD14/CD20/CD34/CD45 (<0.5%) in hADSCs (B). hADSCs obtained from patients of different ages (25 and 48 years old) were differentiated into osteoblasts and adipocytes, which were detected by alkaline phosphatase and Oil Red O staining, respectively (C). Scale bar = 50 μm. Supplementary Figure 3: rat adipose-derived stem cell adipogenic/osteogenic differentiation. Images from adipocytes pre- and poststained with Oil Red O (A) and osteoblasts stained with NBT/BCIP substrate (B) after differentiation induction of ADSCs from 2-, 9-, and 24-month-old rats and from 2-month-old rats treated with 40 mg/kg/day of CH or saline. Scale bar = 50 μm. Supplementary Figure 4: human adipose-derived stem cell adipogenic/osteogenic differentiation. hADSCs obtained from patients of different ages (25-48 years old) were differentiated into osteoblasts and adipocytes, which were detected by alkaline phosphatase or visual vacuole confirmation (sometimes with Oil Red O staining, depending on the availability of the cells to perform all experiments). Note that not all samples were stained with Oil Red O dye because those implied to perform technical duplicates, which [file 6473279.f1.pdf]
